# *N*-Benzyl-*N*,*N*-dimethyl­octa­decan-1-aminium (2-thioxo-1,3-dithiole-4,5-dithiol­ato-κ^2^ *S* ^4^,*S* ^5^)nickelate(III)

**DOI:** 10.1107/S1600536812016698

**Published:** 2012-04-25

**Authors:** Guang-Xiang Liu

**Affiliations:** a School of Biochemical and Environmental Engineering, Nanjing Xiaozhuang University, Nanjing 211171, People’s Republic of China

## Abstract

The asymmtric unit of the title compound, (C_27_H_50_N)[Ni(C_3_S_5_)_2_], contains two *N*-benzyl-*N*,*N*-dimethyl­octa­decan-1-aminium cations, [BDA]^+^, and two [Ni(dmit)_2_]^−^ anions (dmit = 2-thioxo-1,3-dithiole-4,5-dithiol­ate). The C_18_ chains in both cations adopt almost ideal extended conformations. The Ni^III^ atoms are coordinated by two *S*,*S*′-bidentate ligands, generating NiS_4_ square planes. Short Ni⋯S [3.734 (2) Å] and S⋯S contacts [3.5438 (15) Å] occur in the crystal structure; if these are considered to be bonding inter­actions, then infinite sheets of anions parallel to (111) arise.

## Related literature

For applications of bis­(dithiol­ate)-metal complexes, see: Cassoux (1999 ▶). For the oxidation of Ni(II) compounds, see: Cassoux *et al.* (1991 ▶). For the synthesis, see: Xue *et al.* (2003 ▶).
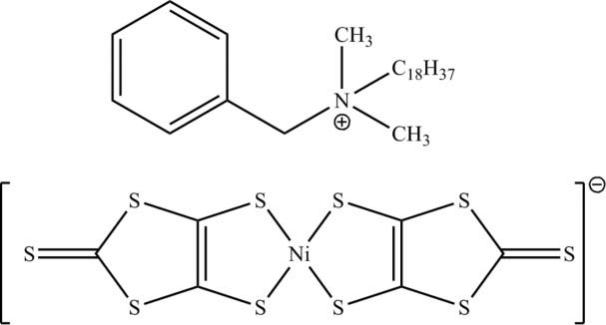


## Experimental

### 

#### Crystal data

(C_27_H_50_N)[Ni(C_3_S_5_)_2_]
*M* *_r_* = 840.05
Triclinic, 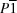

*a* = 12.1626 (16) Å
*b* = 12.2384 (16) Å
*c* = 27.778 (4) Å
α = 80.388 (2)°
β = 84.699 (2)°
γ = 87.431 (2)°
*V* = 4057.4 (9) Å^3^
*Z* = 4
Mo *K*α radiation
μ = 1.02 mm^−1^
*T* = 293 K
0.26 × 0.14 × 0.12 mm

#### Data collection

Bruker SMART APEX CCD diffractometer
Absorption correction: multi-scan (*SADABS*; Bruker, 2000 ▶) *T*_min_ = 0.778, *T*_max_ = 0.888
32319 measured reflections
15041 independent reflections
8322 reflections with *I* > 2σ(*I*)
*R*_int_ = 0.047

#### Refinement

*R*[*F* ^2^ > 2σ(*F* ^2^)] = 0.048
*wR*(*F* ^2^) = 0.128
*S* = 1.01
15041 reflections
817 parameters
H-atom parameters constrained
Δρ_max_ = 0.36 e Å^−3^
Δρ_min_ = −0.32 e Å^−3^

### 

Data collection: *SMART* (Bruker, 2000 ▶); cell refinement: *SAINT* (Bruker, 2000 ▶); data reduction: *SAINT*; program(s) used to solve structure: *SHELXS97* (Sheldrick, 2008 ▶); program(s) used to refine structure: *SHELXL97* (Sheldrick, 2008 ▶); molecular graphics: *SHELXTL* (Sheldrick, 2008 ▶); software used to prepare material for publication: *SHELXTL*.

## Supplementary Material

Crystal structure: contains datablock(s) I, global. DOI: 10.1107/S1600536812016698/hb6730sup1.cif

Structure factors: contains datablock(s) I. DOI: 10.1107/S1600536812016698/hb6730Isup2.hkl

Additional supplementary materials:  crystallographic information; 3D view; checkCIF report

## Figures and Tables

**Table 1:** Selected bond lengths (Å)

Ni1—S2	2.1516 (10)
Ni1—S7	2.1607 (10)
Ni1—S6	2.1631 (10)
Ni1—S1	2.1657 (10)
Ni2—S11	2.1504 (11)
Ni2—S12	2.1591 (11)
Ni2—S17	2.1605 (11)
Ni2—S16	2.1615 (10)

**Fig. 1.**
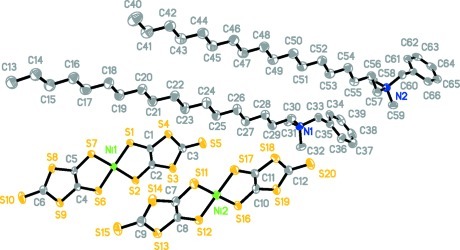
The molecular structure of the title complex, with displacement ellipsoids drawn at the 30% probabilility level.

**Fig. 2.**
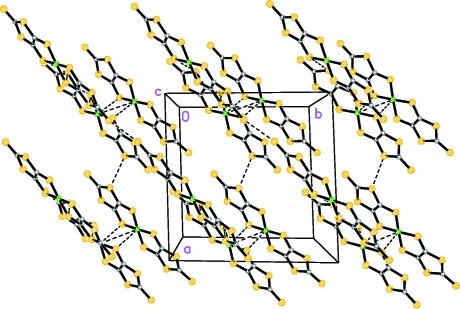
The packing of the [Ni(dmit)2]- anions of (I), showing the S···S and Ni···S contacts as dashed lines.

## supplementary crystallographic information

### Comment

The bis(dithiolate)-metal complexes and their analogues with interesting structures and/or potential applications such as conducting/magnetic or non-linear optical (NLO) materials have been reported in recent years (Cassoux, 1999). We report herein the crystal structure of the title bis-dithiolate-metal complex. In this compound, the Ni(II) cations of NiCl_2_.6H_2_O have been oxidized to Ni(III) cation by I_3_^-^ (Cassoux *et al.*, 1991), the Ni(III) cation is coordinated with two dmit^2-^ anions. As shown in Fig. 1, the asymmetric unit of the title compound contains two crystallographically independent [Ni(dmit)_2_]^-^ anions and two [BDA]^+^ cations. Each Ni(III) ion is coordinated by four S atoms from two dmit ligands to complete a square-planar geometry, with Ni—S bond lengths ranging from 2.1504 (11) to 2.1657 (10) Å. Some of the [Ni(dmit)_2_]^-^ anions are almost parallel each other with the shortest Ni···S distance of 3.734 (2) Å (Ni1—S1^i^) [symmetry code: (i) -*x*, 1 - *y*, -*z*], indicating the existence of the Ni···S interactions. Adjacent [Ni(dmit)_2_]^-^ anions are associated together through such Ni···S interactions result in a dimer. The dimers linked together through S···S interactions forming a two-dimensional layer structure, as depicted in Fig 2. The shortest S···S distance in (I) is equal to the sum of the van der Waals radii and is much larger than that in an analogue with a smaller planar cation, namely *N*-methylpyridinium (Xue *et al.*, 2003); *i.e.* a large cation appears to weaken the intermolecular interaction.

### Experimental

4,5-Di(thiobenzoyl)-1,3-dithiole-2-thione (812 mg, 2 mmol) was suspended in methanol (10 ml). Sodium methoxide in methanol (prepared form 184 mg of sodium in 10 ml of methanol) was added to the above mixture under argon atmosphere at room temperature from 30 min to give a dark red solution. To this solution, NiCl_2_.6H_2_O (238 mg, 1 mmol) was added. After 30 min, a solution of I_2_ (127 mg, 1 mmol) and NaI (150 mg, 1 mmol) in methanol (20 ml) was added (the monoanionic [Ni(dmit)_2_]^-^ are obtained from the dianionic [Ni(dmit)_2_]^2-^ by I_3_^-^ oxidation). After another 10 min, a solution of *N*-benzyl-*N*,*N*-dimethyloctadecan-1-aminium bromide [(BDA)Br] (467 mg, 1 mmol) in methanol (20 ml) was added to the reaction mixture. The solution was stirred for 30 min and cooled in a refrigerator overnight. The resultant dark green crystal was collected by filtration, and purified by recrystallization using a mixed solvent of acetonitrile and benzene.

### Refinement

H atoms were positioned geometrically, with C—H = 0.93, 0.97 and 0.96 Å for aromatic, methylene and methyl H atoms, respectively, and constrained to ride on their parent atoms, with *U*_iso_(H) = xUeq(C), where *x* = 1.5 for methyl H and *x* = 1.2 for all other H atoms.

### Crystal data

**Table:**

(C_27_H_50_N)[Ni(C_3_S_5_)_2_]	*Z* = 4
*M**_r_* = 840.05	*F*(000) = 1772
Triclinic, *P*1	*D*_x_ = 1.375 Mg m^−^^3^
Hall symbol: -P 1	Mo *K*α radiation, λ = 0.71073 Å
*a* = 12.1626 (16) Å	Cell parameters from 4822 reflections
*b* = 12.2384 (16) Å	θ = 2.3–25.0°
*c* = 27.778 (4) Å	µ = 1.02 mm^−^^1^
α = 80.388 (2)°	*T* = 293 K
β = 84.699 (2)°	Pillar, dark green
γ = 87.431 (2)°	0.26 × 0.14 × 0.12 mm
*V* = 4057.4 (9) Å^3^	

### Data collection

**Table:**

Bruker SMART APEX CCD diffractometer	15041 independent reflections
Radiation source: sealed tube	8322 reflections with *I* > 2σ(*I*)
Graphite monochromator	*R*_int_ = 0.047
phi and ω scans	θ_max_ = 25.5°, θ_min_ = 2.3°
Absorption correction: multi-scan (*SADABS*; Bruker, 2000)	*h* = −14→14
*T*_min_ = 0.778, *T*_max_ = 0.888	*k* = −14→14
32319 measured reflections	*l* = −33→33

### Refinement

**Table:**

Refinement on *F*^2^	Primary atom site location: structure-invariant direct methods
Least-squares matrix: full	Secondary atom site location: difference Fourier map
*R*[*F*^2^ > 2σ(*F*^2^)] = 0.048	Hydrogen site location: inferred from neighbouring sites
*wR*(*F*^2^) = 0.128	H-atom parameters constrained
*S* = 1.01	*w* = 1/[σ^2^(*F*_o_^2^) + (0.0429*P*)^2^] where *P* = (*F*_o_^2^ + 2*F*_c_^2^)/3
15041 reflections	(Δ/σ)_max_ = 0.001
817 parameters	Δρ_max_ = 0.36 e Å^−^^3^
0 restraints	Δρ_min_ = −0.32 e Å^−^^3^

### Special details

**Table:**

Geometry. All e.s.d.'s (except the e.s.d. in the dihedral angle between two l.s. planes) are estimated using the full covariance matrix. The cell e.s.d.'s are taken into account individually in the estimation of e.s.d.'s in distances, angles and torsion angles; correlations between e.s.d.'s in cell parameters are only used when they are defined by crystal symmetry. An approximate (isotropic) treatment of cell e.s.d.'s is used for estimating e.s.d.'s involving l.s. planes.
Refinement. Refinement of *F*^2^ against ALL reflections. The weighted *R*-factor *wR* and goodness of fit *S* are based on *F*^2^, conventional *R*-factors *R* are based on *F*, with *F* set to zero for negative *F*^2^. The threshold expression of *F*^2^ > σ(*F*^2^) is used only for calculating *R*-factors(gt) *etc*. and is not relevant to the choice of reflections for refinement. *R*-factors based on *F*^2^ are statistically about twice as large as those based on *F*, and *R*- factors based on ALL data will be even larger.

### Fractional atomic coordinates and isotropic or equivalent isotropic displacement parameters (Å^2^)

**Table:**

	*x*	*y*	*z*	*U*_iso_*/*U*_eq_	
Ni1	0.05179 (4)	0.35723 (4)	0.032575 (16)	0.04308 (14)	
Ni2	0.69111 (4)	0.10274 (4)	0.254025 (17)	0.05030 (15)	
S1	0.08628 (8)	0.48570 (8)	0.07358 (4)	0.0545 (3)	
S2	0.19929 (9)	0.38608 (8)	−0.01624 (4)	0.0590 (3)	
S3	0.37592 (9)	0.55667 (9)	−0.02434 (4)	0.0631 (3)	
S4	0.27316 (9)	0.64806 (8)	0.05830 (4)	0.0598 (3)	
S5	0.48282 (10)	0.74269 (10)	0.00753 (4)	0.0778 (4)	
S6	0.02245 (8)	0.22901 (8)	−0.00930 (3)	0.0537 (3)	
S7	−0.09346 (8)	0.31986 (8)	0.08262 (3)	0.0520 (3)	
S8	−0.26307 (8)	0.14141 (9)	0.08832 (4)	0.0582 (3)	
S9	−0.15202 (9)	0.05522 (8)	0.00610 (4)	0.0607 (3)	
S10	−0.35037 (11)	−0.05852 (11)	0.05959 (5)	0.0867 (4)	
S11	0.55325 (9)	0.05210 (10)	0.30595 (4)	0.0687 (3)	
S12	0.65622 (8)	−0.01272 (8)	0.20693 (4)	0.0584 (3)	
S13	0.47558 (9)	−0.17986 (9)	0.21650 (4)	0.0698 (3)	
S14	0.38445 (9)	−0.12578 (10)	0.30928 (4)	0.0716 (3)	
S15	0.27330 (11)	−0.29187 (10)	0.26532 (5)	0.0878 (4)	
S16	0.82672 (8)	0.15752 (8)	0.20082 (3)	0.0553 (3)	
S17	0.72739 (9)	0.21521 (9)	0.30230 (4)	0.0673 (3)	
S18	0.91155 (10)	0.37827 (9)	0.29458 (4)	0.0715 (3)	
S19	0.99621 (9)	0.33305 (9)	0.19927 (4)	0.0639 (3)	
S20	1.11358 (11)	0.49132 (10)	0.24523 (5)	0.0823 (4)	
C1	0.2066 (3)	0.5381 (3)	0.04195 (13)	0.0458 (9)	
C2	0.2543 (3)	0.4952 (3)	0.00342 (14)	0.0495 (10)	
C3	0.3832 (3)	0.6548 (3)	0.01319 (13)	0.0539 (10)	
C4	−0.0936 (3)	0.1692 (3)	0.02237 (13)	0.0445 (9)	
C5	−0.1446 (3)	0.2096 (3)	0.06152 (13)	0.0445 (9)	
C6	−0.2612 (3)	0.0408 (3)	0.05143 (13)	0.0559 (10)	
C7	0.4994 (3)	−0.0516 (3)	0.28179 (14)	0.0560 (11)	
C8	0.5432 (3)	−0.0792 (3)	0.23878 (15)	0.0526 (10)	
C9	0.3723 (3)	−0.2051 (3)	0.26411 (15)	0.0648 (12)	
C10	0.8830 (3)	0.2570 (3)	0.22686 (15)	0.0552 (10)	
C11	0.8407 (3)	0.2809 (3)	0.27110 (15)	0.0562 (10)	
C12	1.0123 (3)	0.4058 (3)	0.24629 (14)	0.0602 (11)	
C13	−0.4816 (4)	−0.1271 (4)	0.57662 (17)	0.0990 (17)	
H13A	−0.5311	−0.0880	0.5543	0.148*	
H13B	−0.5216	−0.1509	0.6078	0.148*	
H13C	−0.4494	−0.1905	0.5638	0.148*	
C14	−0.3907 (4)	−0.0509 (4)	0.58300 (17)	0.0916 (16)	
H14A	−0.4241	0.0139	0.5951	0.110*	
H14B	−0.3446	−0.0891	0.6076	0.110*	
C15	−0.3207 (4)	−0.0145 (4)	0.53761 (17)	0.0869 (15)	
H15A	−0.3676	0.0231	0.5132	0.104*	
H15B	−0.2883	−0.0798	0.5257	0.104*	
C16	−0.2275 (4)	0.0626 (4)	0.54168 (16)	0.0806 (14)	
H16A	−0.2589	0.1284	0.5535	0.097*	
H16B	−0.1790	0.0253	0.5655	0.097*	
C17	−0.1607 (4)	0.0967 (4)	0.49352 (16)	0.0817 (14)	
H17A	−0.2099	0.1336	0.4699	0.098*	
H17B	−0.1306	0.0303	0.4818	0.098*	
C18	−0.0667 (4)	0.1725 (4)	0.49489 (16)	0.0794 (14)	
H18A	−0.0175	0.1364	0.5186	0.095*	
H18B	−0.0965	0.2398	0.5059	0.095*	
C19	−0.0007 (4)	0.2033 (4)	0.44582 (16)	0.0769 (13)	
H19A	0.0276	0.1358	0.4345	0.092*	
H19B	−0.0497	0.2407	0.4223	0.092*	
C20	0.0949 (3)	0.2772 (4)	0.44726 (15)	0.0733 (13)	
H20A	0.0662	0.3457	0.4575	0.088*	
H20B	0.1424	0.2409	0.4717	0.088*	
C21	0.1637 (3)	0.3050 (4)	0.39889 (15)	0.0740 (13)	
H21A	0.1168	0.3434	0.3747	0.089*	
H21B	0.1906	0.2364	0.3881	0.089*	
C22	0.2614 (3)	0.3760 (4)	0.40075 (15)	0.0719 (12)	
H22A	0.2339	0.4467	0.4092	0.086*	
H22B	0.3049	0.3402	0.4268	0.086*	
C23	0.3359 (3)	0.3977 (4)	0.35408 (15)	0.0740 (13)	
H23A	0.2925	0.4333	0.3279	0.089*	
H23B	0.3641	0.3272	0.3457	0.089*	
C24	0.4328 (4)	0.4695 (4)	0.35642 (15)	0.0779 (14)	
H24A	0.4751	0.4352	0.3832	0.094*	
H24B	0.4047	0.5409	0.3637	0.094*	
C25	0.5095 (3)	0.4883 (3)	0.30979 (14)	0.0648 (12)	
H25A	0.4664	0.5187	0.2827	0.078*	
H25B	0.5407	0.4172	0.3035	0.078*	
C26	0.6033 (3)	0.5655 (4)	0.31108 (14)	0.0697 (12)	
H26A	0.5726	0.6353	0.3193	0.084*	
H26B	0.6495	0.5329	0.3367	0.084*	
C27	0.6743 (3)	0.5881 (3)	0.26295 (13)	0.0633 (11)	
H27A	0.7085	0.5187	0.2559	0.076*	
H27B	0.6271	0.6159	0.2370	0.076*	
C28	0.7639 (3)	0.6704 (3)	0.26225 (13)	0.0620 (11)	
H28A	0.8151	0.6396	0.2861	0.074*	
H28B	0.7306	0.7376	0.2721	0.074*	
C29	0.8278 (3)	0.6999 (3)	0.21268 (14)	0.0644 (12)	
H29A	0.8604	0.6330	0.2021	0.077*	
H29B	0.7780	0.7337	0.1888	0.077*	
C30	0.9176 (3)	0.7791 (3)	0.21544 (13)	0.0600 (11)	
H30A	0.9675	0.7425	0.2388	0.072*	
H30B	0.8836	0.8423	0.2286	0.072*	
C31	0.9149 (3)	0.8873 (3)	0.13133 (13)	0.0631 (11)	
H31A	0.8754	0.9456	0.1454	0.095*	
H31B	0.8633	0.8388	0.1223	0.095*	
H31C	0.9605	0.9188	0.1027	0.095*	
C32	1.0434 (3)	0.7289 (3)	0.14640 (15)	0.0694 (12)	
H32A	0.9893	0.6807	0.1391	0.104*	
H32B	1.0905	0.6880	0.1694	0.104*	
H32C	1.0870	0.7579	0.1168	0.104*	
C33	1.0686 (3)	0.8990 (3)	0.18233 (13)	0.0603 (11)	
H33A	1.0274	0.9597	0.1947	0.072*	
H33B	1.1068	0.8579	0.2091	0.072*	
C34	1.1540 (3)	0.9476 (3)	0.14287 (14)	0.0532 (10)	
C35	1.2525 (3)	0.8925 (3)	0.13538 (15)	0.0662 (12)	
H35	1.2643	0.8225	0.1534	0.079*	
C36	1.3354 (4)	0.9389 (4)	0.10130 (17)	0.0789 (14)	
H36	1.4023	0.9005	0.0968	0.095*	
C37	1.3180 (5)	1.0408 (4)	0.07455 (16)	0.0792 (15)	
H37	1.3733	1.0717	0.0516	0.095*	
C38	1.2212 (5)	1.0979 (4)	0.08092 (17)	0.0808 (15)	
H38	1.2098	1.1672	0.0621	0.097*	
C39	1.1385 (4)	1.0523 (4)	0.11584 (16)	0.0674 (12)	
H39	1.0728	1.0922	0.1210	0.081*	
C40	−0.0553 (5)	0.3611 (5)	0.6307 (2)	0.128 (2)	
H40A	−0.0117	0.3257	0.6564	0.193*	
H40B	−0.1107	0.3117	0.6257	0.193*	
H40C	−0.0903	0.4274	0.6399	0.193*	
C41	0.0174 (4)	0.3900 (4)	0.58462 (18)	0.0980 (16)	
H41A	−0.0279	0.4258	0.5592	0.118*	
H41B	0.0481	0.3218	0.5748	0.118*	
C42	0.1109 (4)	0.4643 (4)	0.58701 (16)	0.0789 (13)	
H42A	0.1576	0.4280	0.6118	0.095*	
H42B	0.0807	0.5321	0.5974	0.095*	
C43	0.1815 (4)	0.4942 (4)	0.53954 (16)	0.0811 (14)	
H43A	0.2116	0.4260	0.5294	0.097*	
H43B	0.1342	0.5293	0.5148	0.097*	
C44	0.2751 (4)	0.5688 (4)	0.54003 (16)	0.0816 (14)	
H44A	0.3235	0.5335	0.5643	0.098*	
H44B	0.2455	0.6369	0.5504	0.098*	
C45	0.3429 (4)	0.5977 (4)	0.49172 (16)	0.0810 (14)	
H45A	0.3725	0.5297	0.4814	0.097*	
H45B	0.2946	0.6332	0.4674	0.097*	
C46	0.4369 (4)	0.6729 (4)	0.49267 (16)	0.0802 (14)	
H46A	0.4840	0.6381	0.5176	0.096*	
H46B	0.4068	0.7415	0.5024	0.096*	
C47	0.5081 (4)	0.7010 (4)	0.44420 (16)	0.0788 (13)	
H47A	0.5393	0.6327	0.4347	0.095*	
H47B	0.4611	0.7350	0.4191	0.095*	
C48	0.6011 (4)	0.7779 (4)	0.44564 (16)	0.0789 (13)	
H48A	0.5695	0.8475	0.4536	0.095*	
H48B	0.6459	0.7456	0.4719	0.095*	
C49	0.6753 (4)	0.8019 (4)	0.39876 (15)	0.0751 (13)	
H49A	0.6305	0.8329	0.3724	0.090*	
H49B	0.7083	0.7324	0.3911	0.090*	
C50	0.7668 (4)	0.8808 (4)	0.40021 (16)	0.0765 (13)	
H50A	0.8091	0.8520	0.4278	0.092*	
H50B	0.7338	0.9516	0.4059	0.092*	
C51	0.8450 (4)	0.8995 (3)	0.35412 (14)	0.0712 (12)	
H51A	0.8025	0.9286	0.3267	0.085*	
H51B	0.8772	0.8284	0.3484	0.085*	
C52	0.9382 (4)	0.9777 (4)	0.35481 (15)	0.0743 (13)	
H52A	0.9065	1.0494	0.3601	0.089*	
H52B	0.9809	0.9491	0.3822	0.089*	
C53	1.0143 (4)	0.9933 (3)	0.30868 (14)	0.0697 (12)	
H53A	0.9696	1.0135	0.2812	0.084*	
H53B	1.0505	0.9223	0.3054	0.084*	
C54	1.1025 (3)	1.0783 (4)	0.30422 (15)	0.0708 (12)	
H54A	1.0673	1.1500	0.3069	0.085*	
H54B	1.1485	1.0586	0.3313	0.085*	
C55	1.1744 (3)	1.0877 (3)	0.25698 (14)	0.0667 (12)	
H55A	1.1268	1.0994	0.2303	0.080*	
H55B	1.2137	1.0173	0.2560	0.080*	
C56	1.2597 (3)	1.1795 (3)	0.24710 (15)	0.0701 (12)	
H56A	1.2220	1.2514	0.2412	0.084*	
H56B	1.3009	1.1767	0.2756	0.084*	
C57	1.3379 (4)	1.1648 (3)	0.20322 (15)	0.0711 (13)	
H57A	1.2987	1.1287	0.1817	0.085*	
H57B	1.3976	1.1145	0.2145	0.085*	
C58	1.3050 (4)	1.3378 (3)	0.14485 (16)	0.0792 (14)	
H58A	1.3407	1.3956	0.1219	0.119*	
H58B	1.2536	1.3702	0.1672	0.119*	
H58C	1.2662	1.2922	0.1275	0.119*	
C59	1.4756 (3)	1.2293 (3)	0.13660 (15)	0.0750 (13)	
H59A	1.4419	1.1839	0.1174	0.113*	
H59B	1.5321	1.1867	0.1540	0.113*	
H59C	1.5078	1.2920	0.1154	0.113*	
C60	1.4428 (3)	1.3343 (3)	0.20586 (14)	0.0636 (11)	
H60A	1.3856	1.3608	0.2282	0.076*	
H60B	1.4924	1.2849	0.2254	0.076*	
C61	1.5070 (4)	1.4322 (3)	0.17860 (13)	0.0550 (10)	
C62	1.4553 (4)	1.5329 (4)	0.16524 (15)	0.0685 (12)	
H62	1.3803	1.5425	0.1741	0.082*	
C63	1.5138 (5)	1.6200 (4)	0.13867 (16)	0.0741 (13)	
H63	1.4775	1.6877	0.1294	0.089*	
C64	1.6239 (5)	1.6089 (4)	0.12581 (15)	0.0765 (14)	
H64	1.6624	1.6682	0.1077	0.092*	
C65	1.6780 (4)	1.5084 (4)	0.13985 (16)	0.0727 (13)	
H65	1.7534	1.4999	0.1316	0.087*	
C66	1.6190 (4)	1.4205 (3)	0.16644 (14)	0.0636 (11)	
H66	1.6552	1.3530	0.1762	0.076*	
N1	0.9864 (2)	0.8228 (2)	0.16809 (10)	0.0490 (8)	
N2	1.3894 (3)	1.2690 (3)	0.17266 (11)	0.0577 (9)	

### Atomic displacement parameters (Å^2^)

**Table:**

	*U*^11^	*U*^22^	*U*^33^	*U*^12^	*U*^13^	*U*^23^
Ni1	0.0433 (3)	0.0436 (3)	0.0423 (3)	−0.0071 (2)	0.0000 (2)	−0.0074 (2)
Ni2	0.0481 (3)	0.0541 (3)	0.0489 (3)	−0.0058 (2)	−0.0017 (2)	−0.0093 (2)
S1	0.0518 (6)	0.0551 (6)	0.0590 (6)	−0.0111 (5)	0.0069 (5)	−0.0201 (5)
S2	0.0594 (7)	0.0645 (7)	0.0563 (6)	−0.0255 (5)	0.0151 (5)	−0.0241 (5)
S3	0.0615 (7)	0.0715 (7)	0.0579 (7)	−0.0288 (6)	0.0084 (5)	−0.0151 (6)
S4	0.0631 (7)	0.0540 (6)	0.0662 (7)	−0.0150 (5)	−0.0015 (6)	−0.0198 (5)
S5	0.0853 (9)	0.0785 (8)	0.0710 (8)	−0.0465 (7)	−0.0046 (6)	−0.0059 (6)
S6	0.0531 (6)	0.0619 (6)	0.0488 (6)	−0.0193 (5)	0.0104 (5)	−0.0200 (5)
S7	0.0495 (6)	0.0576 (6)	0.0511 (6)	−0.0105 (5)	0.0085 (5)	−0.0201 (5)
S8	0.0436 (6)	0.0780 (7)	0.0531 (6)	−0.0183 (5)	0.0053 (5)	−0.0123 (5)
S9	0.0614 (7)	0.0656 (7)	0.0597 (7)	−0.0221 (6)	0.0037 (5)	−0.0228 (6)
S10	0.0811 (9)	0.0988 (9)	0.0826 (9)	−0.0529 (8)	−0.0003 (7)	−0.0119 (7)
S11	0.0667 (8)	0.0880 (8)	0.0548 (7)	−0.0230 (6)	0.0095 (6)	−0.0240 (6)
S12	0.0528 (7)	0.0647 (7)	0.0595 (7)	−0.0120 (5)	0.0076 (5)	−0.0193 (5)
S13	0.0619 (8)	0.0692 (7)	0.0832 (8)	−0.0139 (6)	−0.0026 (6)	−0.0258 (6)
S14	0.0611 (8)	0.0866 (8)	0.0648 (7)	−0.0230 (6)	0.0034 (6)	−0.0047 (6)
S15	0.0737 (9)	0.0810 (9)	0.1069 (10)	−0.0317 (7)	−0.0152 (7)	0.0021 (8)
S16	0.0554 (7)	0.0647 (7)	0.0485 (6)	−0.0156 (5)	−0.0005 (5)	−0.0157 (5)
S17	0.0665 (8)	0.0761 (8)	0.0627 (7)	−0.0152 (6)	0.0078 (6)	−0.0255 (6)
S18	0.0752 (8)	0.0688 (7)	0.0786 (8)	−0.0104 (6)	−0.0094 (6)	−0.0317 (6)
S19	0.0586 (7)	0.0724 (7)	0.0622 (7)	−0.0209 (6)	−0.0065 (5)	−0.0098 (6)
S20	0.0809 (9)	0.0668 (8)	0.1041 (10)	−0.0231 (7)	−0.0249 (7)	−0.0133 (7)
C1	0.048 (2)	0.040 (2)	0.049 (2)	−0.0065 (18)	−0.0109 (19)	−0.0029 (18)
C2	0.043 (2)	0.048 (2)	0.054 (2)	−0.0156 (18)	0.0002 (19)	0.0022 (19)
C3	0.063 (3)	0.049 (2)	0.047 (2)	−0.010 (2)	−0.009 (2)	0.0026 (19)
C4	0.045 (2)	0.046 (2)	0.043 (2)	−0.0063 (18)	−0.0103 (18)	−0.0051 (18)
C5	0.033 (2)	0.052 (2)	0.048 (2)	−0.0062 (17)	−0.0065 (17)	−0.0005 (18)
C6	0.056 (3)	0.067 (3)	0.046 (2)	−0.011 (2)	−0.0079 (19)	−0.006 (2)
C7	0.049 (3)	0.063 (3)	0.051 (3)	−0.004 (2)	−0.003 (2)	0.005 (2)
C8	0.042 (2)	0.049 (2)	0.067 (3)	−0.0038 (18)	−0.013 (2)	−0.007 (2)
C9	0.055 (3)	0.066 (3)	0.069 (3)	0.004 (2)	−0.014 (2)	0.005 (2)
C10	0.050 (3)	0.053 (2)	0.062 (3)	−0.007 (2)	−0.013 (2)	0.002 (2)
C11	0.053 (3)	0.050 (2)	0.068 (3)	−0.004 (2)	−0.014 (2)	−0.012 (2)
C12	0.058 (3)	0.053 (2)	0.069 (3)	0.000 (2)	−0.014 (2)	−0.006 (2)
C13	0.105 (4)	0.114 (4)	0.082 (4)	−0.032 (3)	0.010 (3)	−0.028 (3)
C14	0.093 (4)	0.104 (4)	0.077 (3)	−0.019 (3)	0.012 (3)	−0.017 (3)
C15	0.089 (4)	0.087 (3)	0.081 (4)	−0.011 (3)	0.005 (3)	−0.010 (3)
C16	0.074 (3)	0.084 (3)	0.079 (3)	−0.008 (3)	0.006 (3)	−0.006 (3)
C17	0.086 (4)	0.076 (3)	0.079 (3)	−0.005 (3)	0.010 (3)	−0.010 (3)
C18	0.067 (3)	0.086 (3)	0.083 (3)	−0.012 (3)	0.006 (3)	−0.011 (3)
C19	0.069 (3)	0.085 (3)	0.074 (3)	−0.008 (3)	0.007 (2)	−0.010 (3)
C20	0.061 (3)	0.080 (3)	0.077 (3)	−0.005 (2)	0.004 (2)	−0.013 (3)
C21	0.067 (3)	0.086 (3)	0.069 (3)	−0.011 (3)	−0.001 (2)	−0.014 (3)
C22	0.063 (3)	0.086 (3)	0.065 (3)	−0.010 (3)	0.002 (2)	−0.009 (2)
C23	0.069 (3)	0.091 (3)	0.061 (3)	−0.014 (3)	−0.009 (2)	−0.004 (3)
C24	0.072 (3)	0.107 (4)	0.055 (3)	−0.019 (3)	−0.003 (2)	−0.010 (3)
C25	0.063 (3)	0.080 (3)	0.051 (3)	−0.007 (2)	−0.008 (2)	−0.008 (2)
C26	0.068 (3)	0.092 (3)	0.048 (3)	−0.020 (3)	−0.005 (2)	−0.007 (2)
C27	0.062 (3)	0.076 (3)	0.050 (3)	−0.007 (2)	−0.004 (2)	−0.006 (2)
C28	0.065 (3)	0.071 (3)	0.050 (3)	−0.007 (2)	−0.005 (2)	−0.007 (2)
C29	0.070 (3)	0.074 (3)	0.050 (3)	−0.013 (2)	−0.002 (2)	−0.011 (2)
C30	0.065 (3)	0.072 (3)	0.043 (2)	−0.003 (2)	0.001 (2)	−0.012 (2)
C31	0.063 (3)	0.074 (3)	0.050 (3)	0.003 (2)	−0.010 (2)	−0.004 (2)
C32	0.069 (3)	0.064 (3)	0.073 (3)	0.010 (2)	0.011 (2)	−0.019 (2)
C33	0.053 (3)	0.079 (3)	0.052 (3)	−0.010 (2)	−0.005 (2)	−0.017 (2)
C34	0.057 (3)	0.055 (3)	0.048 (2)	−0.001 (2)	−0.009 (2)	−0.008 (2)
C35	0.059 (3)	0.062 (3)	0.071 (3)	−0.003 (2)	0.002 (2)	0.004 (2)
C36	0.063 (3)	0.083 (3)	0.090 (4)	−0.013 (3)	0.012 (3)	−0.020 (3)
C37	0.096 (4)	0.087 (4)	0.056 (3)	−0.043 (3)	−0.007 (3)	−0.006 (3)
C38	0.116 (5)	0.063 (3)	0.066 (3)	−0.028 (3)	−0.038 (3)	0.008 (3)
C39	0.071 (3)	0.066 (3)	0.073 (3)	−0.001 (2)	−0.022 (3)	−0.023 (3)
C40	0.121 (5)	0.126 (5)	0.120 (5)	0.002 (4)	0.034 (4)	0.006 (4)
C41	0.101 (4)	0.092 (4)	0.094 (4)	−0.001 (3)	0.004 (3)	0.000 (3)
C42	0.082 (4)	0.083 (3)	0.072 (3)	0.001 (3)	−0.007 (3)	−0.014 (3)
C43	0.093 (4)	0.077 (3)	0.071 (3)	0.001 (3)	−0.007 (3)	−0.007 (3)
C44	0.084 (4)	0.082 (3)	0.078 (3)	0.001 (3)	−0.008 (3)	−0.014 (3)
C45	0.087 (4)	0.078 (3)	0.077 (3)	−0.003 (3)	−0.003 (3)	−0.011 (3)
C46	0.076 (3)	0.084 (3)	0.081 (3)	−0.002 (3)	−0.005 (3)	−0.015 (3)
C47	0.086 (4)	0.078 (3)	0.072 (3)	−0.002 (3)	−0.003 (3)	−0.012 (3)
C48	0.073 (3)	0.089 (3)	0.074 (3)	−0.006 (3)	0.002 (3)	−0.014 (3)
C49	0.081 (3)	0.076 (3)	0.065 (3)	0.002 (3)	−0.003 (3)	−0.006 (2)
C50	0.073 (3)	0.083 (3)	0.071 (3)	0.001 (3)	−0.002 (3)	−0.012 (3)
C51	0.080 (3)	0.076 (3)	0.054 (3)	0.005 (3)	−0.001 (2)	−0.005 (2)
C52	0.071 (3)	0.090 (3)	0.061 (3)	−0.002 (3)	−0.003 (2)	−0.014 (3)
C53	0.079 (3)	0.072 (3)	0.054 (3)	0.001 (3)	0.001 (2)	−0.003 (2)
C54	0.073 (3)	0.081 (3)	0.061 (3)	−0.008 (3)	−0.008 (2)	−0.013 (2)
C55	0.076 (3)	0.062 (3)	0.061 (3)	0.000 (2)	−0.005 (2)	−0.008 (2)
C56	0.068 (3)	0.076 (3)	0.066 (3)	−0.008 (2)	−0.002 (2)	−0.014 (2)
C57	0.073 (3)	0.066 (3)	0.074 (3)	−0.014 (2)	0.008 (3)	−0.014 (2)
C58	0.078 (3)	0.079 (3)	0.086 (3)	0.005 (3)	−0.033 (3)	−0.017 (3)
C59	0.082 (3)	0.077 (3)	0.069 (3)	−0.003 (3)	0.011 (3)	−0.027 (2)
C60	0.061 (3)	0.080 (3)	0.052 (3)	−0.001 (2)	−0.007 (2)	−0.016 (2)
C61	0.061 (3)	0.063 (3)	0.045 (2)	0.001 (2)	−0.007 (2)	−0.018 (2)
C62	0.075 (3)	0.072 (3)	0.064 (3)	0.006 (3)	−0.014 (2)	−0.027 (3)
C63	0.107 (4)	0.062 (3)	0.057 (3)	0.003 (3)	−0.010 (3)	−0.022 (2)
C64	0.112 (4)	0.071 (3)	0.050 (3)	−0.024 (3)	0.007 (3)	−0.023 (2)
C65	0.068 (3)	0.086 (4)	0.069 (3)	−0.015 (3)	0.010 (2)	−0.033 (3)
C66	0.072 (3)	0.061 (3)	0.060 (3)	0.002 (2)	−0.011 (2)	−0.017 (2)
N1	0.0452 (19)	0.059 (2)	0.0420 (18)	0.0037 (16)	0.0006 (15)	−0.0088 (16)
N2	0.055 (2)	0.069 (2)	0.050 (2)	−0.0030 (18)	−0.0023 (17)	−0.0124 (18)

### Geometric parameters (Å, º)

**Table:**

Ni1—S2	2.1516 (10)	C31—H31C	0.9600
Ni1—S7	2.1607 (10)	C32—N1	1.499 (4)
Ni1—S6	2.1631 (10)	C32—H32A	0.9600
Ni1—S1	2.1657 (10)	C32—H32B	0.9600
Ni2—S11	2.1504 (11)	C32—H32C	0.9600
Ni2—S12	2.1591 (11)	C33—C34	1.505 (5)
Ni2—S17	2.1605 (11)	C33—N1	1.519 (4)
Ni2—S16	2.1615 (10)	C33—H33A	0.9700
S1—C1	1.727 (4)	C33—H33B	0.9700
S2—C2	1.709 (4)	C34—C35	1.364 (5)
S3—C3	1.727 (4)	C34—C39	1.387 (5)
S3—C2	1.745 (3)	C35—C36	1.390 (5)
S4—C3	1.742 (4)	C35—H35	0.9300
S4—C1	1.744 (3)	C36—C37	1.359 (6)
S5—C3	1.635 (4)	C36—H36	0.9300
S6—C4	1.718 (3)	C37—C38	1.355 (6)
S7—C5	1.720 (3)	C37—H37	0.9300
S8—C6	1.726 (4)	C38—C39	1.398 (6)
S8—C5	1.740 (3)	C38—H38	0.9300
S9—C4	1.736 (3)	C39—H39	0.9300
S9—C6	1.737 (4)	C40—C41	1.488 (6)
S10—C6	1.639 (4)	C40—H40A	0.9600
S11—C7	1.712 (4)	C40—H40B	0.9600
S12—C8	1.723 (4)	C40—H40C	0.9600
S13—C9	1.736 (4)	C41—C42	1.500 (6)
S13—C8	1.737 (4)	C41—H41A	0.9700
S14—C9	1.730 (4)	C41—H41B	0.9700
S14—C7	1.746 (4)	C42—C43	1.506 (5)
S15—C9	1.635 (4)	C42—H42A	0.9700
S16—C10	1.713 (4)	C42—H42B	0.9700
S17—C11	1.715 (4)	C43—C44	1.493 (5)
S18—C12	1.731 (4)	C43—H43A	0.9700
S18—C11	1.745 (4)	C43—H43B	0.9700
S19—C12	1.729 (4)	C44—C45	1.506 (5)
S19—C10	1.741 (4)	C44—H44A	0.9700
S20—C12	1.647 (4)	C44—H44B	0.9700
C1—C2	1.343 (5)	C45—C46	1.504 (5)
C4—C5	1.357 (5)	C45—H45A	0.9700
C7—C8	1.351 (5)	C45—H45B	0.9700
C10—C11	1.360 (5)	C46—C47	1.529 (5)
C13—C14	1.518 (5)	C46—H46A	0.9700
C13—H13A	0.9600	C46—H46B	0.9700
C13—H13B	0.9600	C47—C48	1.510 (5)
C13—H13C	0.9600	C47—H47A	0.9700
C14—C15	1.470 (5)	C47—H47B	0.9700
C14—H14A	0.9700	C48—C49	1.509 (5)
C14—H14B	0.9700	C48—H48A	0.9700
C15—C16	1.531 (5)	C48—H48B	0.9700
C15—H15A	0.9700	C49—C50	1.513 (5)
C15—H15B	0.9700	C49—H49A	0.9700
C16—C17	1.506 (5)	C49—H49B	0.9700
C16—H16A	0.9700	C50—C51	1.514 (5)
C16—H16B	0.9700	C50—H50A	0.9700
C17—C18	1.511 (5)	C50—H50B	0.9700
C17—H17A	0.9700	C51—C52	1.519 (5)
C17—H17B	0.9700	C51—H51A	0.9700
C18—C19	1.516 (5)	C51—H51B	0.9700
C18—H18A	0.9700	C52—C53	1.500 (5)
C18—H18B	0.9700	C52—H52A	0.9700
C19—C20	1.513 (5)	C52—H52B	0.9700
C19—H19A	0.9700	C53—C54	1.510 (5)
C19—H19B	0.9700	C53—H53A	0.9700
C20—C21	1.512 (5)	C53—H53B	0.9700
C20—H20A	0.9700	C54—C55	1.499 (5)
C20—H20B	0.9700	C54—H54A	0.9700
C21—C22	1.513 (5)	C54—H54B	0.9700
C21—H21A	0.9700	C55—C56	1.537 (5)
C21—H21B	0.9700	C55—H55A	0.9700
C22—C23	1.504 (5)	C55—H55B	0.9700
C22—H22A	0.9700	C56—C57	1.506 (5)
C22—H22B	0.9700	C56—H56A	0.9700
C23—C24	1.514 (5)	C56—H56B	0.9700
C23—H23A	0.9700	C57—N2	1.533 (4)
C23—H23B	0.9700	C57—H57A	0.9700
C24—C25	1.515 (5)	C57—H57B	0.9700
C24—H24A	0.9700	C58—N2	1.488 (5)
C24—H24B	0.9700	C58—H58A	0.9600
C25—C26	1.522 (5)	C58—H58B	0.9600
C25—H25A	0.9700	C58—H58C	0.9600
C25—H25B	0.9700	C59—N2	1.503 (4)
C26—C27	1.514 (5)	C59—H59A	0.9600
C26—H26A	0.9700	C59—H59B	0.9600
C26—H26B	0.9700	C59—H59C	0.9600
C27—C28	1.514 (5)	C60—C61	1.513 (5)
C27—H27A	0.9700	C60—N2	1.521 (4)
C27—H27B	0.9700	C60—H60A	0.9700
C28—C29	1.514 (5)	C60—H60B	0.9700
C28—H28A	0.9700	C61—C62	1.368 (5)
C28—H28B	0.9700	C61—C66	1.380 (5)
C29—C30	1.508 (5)	C62—C63	1.378 (5)
C29—H29A	0.9700	C62—H62	0.9300
C29—H29B	0.9700	C63—C64	1.360 (6)
C30—N1	1.522 (4)	C63—H63	0.9300
C30—H30A	0.9700	C64—C65	1.382 (6)
C30—H30B	0.9700	C64—H64	0.9300
C31—N1	1.501 (4)	C65—C66	1.387 (5)
C31—H31A	0.9600	C65—H65	0.9300
C31—H31B	0.9600	C66—H66	0.9300
			
S2—Ni1—S7	177.16 (5)	H32A—C32—H32C	109.5
S2—Ni1—S6	85.19 (4)	H32B—C32—H32C	109.5
S7—Ni1—S6	93.30 (4)	C34—C33—N1	117.0 (3)
S2—Ni1—S1	93.15 (4)	C34—C33—H33A	108.1
S7—Ni1—S1	88.33 (4)	N1—C33—H33A	108.1
S6—Ni1—S1	178.28 (4)	C34—C33—H33B	108.1
S11—Ni2—S12	92.97 (4)	N1—C33—H33B	108.1
S11—Ni2—S17	86.67 (4)	H33A—C33—H33B	107.3
S12—Ni2—S17	178.72 (5)	C35—C34—C39	118.4 (4)
S11—Ni2—S16	178.34 (5)	C35—C34—C33	120.2 (4)
S12—Ni2—S16	87.17 (4)	C39—C34—C33	121.2 (4)
S17—Ni2—S16	93.22 (4)	C34—C35—C36	121.3 (4)
C1—S1—Ni1	101.54 (13)	C34—C35—H35	119.4
C2—S2—Ni1	102.33 (13)	C36—C35—H35	119.4
C3—S3—C2	98.11 (18)	C37—C36—C35	119.5 (5)
C3—S4—C1	97.51 (18)	C37—C36—H36	120.2
C4—S6—Ni1	102.04 (12)	C35—C36—H36	120.2
C5—S7—Ni1	101.94 (12)	C38—C37—C36	120.8 (5)
C6—S8—C5	98.40 (17)	C38—C37—H37	119.6
C4—S9—C6	97.65 (17)	C36—C37—H37	119.6
C7—S11—Ni2	102.71 (14)	C37—C38—C39	119.7 (4)
C8—S12—Ni2	102.07 (14)	C37—C38—H38	120.1
C9—S13—C8	97.8 (2)	C39—C38—H38	120.1
C9—S14—C7	97.88 (19)	C34—C39—C38	120.2 (4)
C10—S16—Ni2	102.23 (14)	C34—C39—H39	119.9
C11—S17—Ni2	101.98 (14)	C38—C39—H39	119.9
C12—S18—C11	97.58 (19)	C41—C40—H40A	109.5
C12—S19—C10	97.68 (19)	C41—C40—H40B	109.5
C2—C1—S1	121.3 (3)	H40A—C40—H40B	109.5
C2—C1—S4	116.4 (3)	C41—C40—H40C	109.5
S1—C1—S4	122.3 (2)	H40A—C40—H40C	109.5
C1—C2—S2	121.6 (3)	H40B—C40—H40C	109.5
C1—C2—S3	115.8 (3)	C40—C41—C42	115.9 (5)
S2—C2—S3	122.6 (2)	C40—C41—H41A	108.3
S5—C3—S3	123.9 (2)	C42—C41—H41A	108.3
S5—C3—S4	123.9 (2)	C40—C41—H41B	108.3
S3—C3—S4	112.2 (2)	C42—C41—H41B	108.3
C5—C4—S6	121.2 (3)	H41A—C41—H41B	107.4
C5—C4—S9	116.6 (3)	C41—C42—C43	114.8 (4)
S6—C4—S9	122.2 (2)	C41—C42—H42A	108.6
C4—C5—S7	121.5 (3)	C43—C42—H42A	108.6
C4—C5—S8	115.2 (3)	C41—C42—H42B	108.6
S7—C5—S8	123.4 (2)	C43—C42—H42B	108.6
S10—C6—S8	123.5 (2)	H42A—C42—H42B	107.6
S10—C6—S9	124.3 (2)	C44—C43—C42	116.7 (4)
S8—C6—S9	112.1 (2)	C44—C43—H43A	108.1
C8—C7—S11	121.0 (3)	C42—C43—H43A	108.1
C8—C7—S14	115.6 (3)	C44—C43—H43B	108.1
S11—C7—S14	123.3 (2)	C42—C43—H43B	108.1
C7—C8—S12	121.2 (3)	H43A—C43—H43B	107.3
C7—C8—S13	116.3 (3)	C43—C44—C45	115.0 (4)
S12—C8—S13	122.4 (2)	C43—C44—H44A	108.5
S15—C9—S14	124.0 (3)	C45—C44—H44A	108.5
S15—C9—S13	123.7 (3)	C43—C44—H44B	108.5
S14—C9—S13	112.3 (2)	C45—C44—H44B	108.5
C11—C10—S16	121.0 (3)	H44A—C44—H44B	107.5
C11—C10—S19	115.9 (3)	C46—C45—C44	114.6 (4)
S16—C10—S19	123.0 (2)	C46—C45—H45A	108.6
C10—C11—S17	121.5 (3)	C44—C45—H45A	108.6
C10—C11—S18	115.8 (3)	C46—C45—H45B	108.6
S17—C11—S18	122.6 (2)	C44—C45—H45B	108.6
S20—C12—S19	123.4 (2)	H45A—C45—H45B	107.6
S20—C12—S18	123.8 (2)	C45—C46—C47	115.2 (4)
S19—C12—S18	112.9 (2)	C45—C46—H46A	108.5
C14—C13—H13A	109.5	C47—C46—H46A	108.5
C14—C13—H13B	109.5	C45—C46—H46B	108.5
H13A—C13—H13B	109.5	C47—C46—H46B	108.5
C14—C13—H13C	109.5	H46A—C46—H46B	107.5
H13A—C13—H13C	109.5	C48—C47—C46	114.5 (4)
H13B—C13—H13C	109.5	C48—C47—H47A	108.6
C15—C14—C13	113.6 (4)	C46—C47—H47A	108.6
C15—C14—H14A	108.9	C48—C47—H47B	108.6
C13—C14—H14A	108.9	C46—C47—H47B	108.6
C15—C14—H14B	108.9	H47A—C47—H47B	107.6
C13—C14—H14B	108.9	C49—C48—C47	115.0 (4)
H14A—C14—H14B	107.7	C49—C48—H48A	108.5
C14—C15—C16	116.1 (4)	C47—C48—H48A	108.5
C14—C15—H15A	108.3	C49—C48—H48B	108.5
C16—C15—H15A	108.3	C47—C48—H48B	108.5
C14—C15—H15B	108.3	H48A—C48—H48B	107.5
C16—C15—H15B	108.3	C48—C49—C50	114.6 (4)
H15A—C15—H15B	107.4	C48—C49—H49A	108.6
C17—C16—C15	112.6 (4)	C50—C49—H49A	108.6
C17—C16—H16A	109.1	C48—C49—H49B	108.6
C15—C16—H16A	109.1	C50—C49—H49B	108.6
C17—C16—H16B	109.1	H49A—C49—H49B	107.6
C15—C16—H16B	109.1	C49—C50—C51	114.4 (4)
H16A—C16—H16B	107.8	C49—C50—H50A	108.7
C16—C17—C18	115.4 (4)	C51—C50—H50A	108.7
C16—C17—H17A	108.4	C49—C50—H50B	108.7
C18—C17—H17A	108.4	C51—C50—H50B	108.7
C16—C17—H17B	108.4	H50A—C50—H50B	107.6
C18—C17—H17B	108.4	C50—C51—C52	115.5 (4)
H17A—C17—H17B	107.5	C50—C51—H51A	108.4
C17—C18—C19	113.6 (4)	C52—C51—H51A	108.4
C17—C18—H18A	108.8	C50—C51—H51B	108.4
C19—C18—H18A	108.8	C52—C51—H51B	108.4
C17—C18—H18B	108.8	H51A—C51—H51B	107.5
C19—C18—H18B	108.8	C53—C52—C51	113.8 (4)
H18A—C18—H18B	107.7	C53—C52—H52A	108.8
C20—C19—C18	113.8 (4)	C51—C52—H52A	108.8
C20—C19—H19A	108.8	C53—C52—H52B	108.8
C18—C19—H19A	108.8	C51—C52—H52B	108.8
C20—C19—H19B	108.8	H52A—C52—H52B	107.7
C18—C19—H19B	108.8	C52—C53—C54	117.3 (4)
H19A—C19—H19B	107.7	C52—C53—H53A	108.0
C21—C20—C19	114.2 (4)	C54—C53—H53A	108.0
C21—C20—H20A	108.7	C52—C53—H53B	108.0
C19—C20—H20A	108.7	C54—C53—H53B	108.0
C21—C20—H20B	108.7	H53A—C53—H53B	107.2
C19—C20—H20B	108.7	C55—C54—C53	113.3 (3)
H20A—C20—H20B	107.6	C55—C54—H54A	108.9
C20—C21—C22	114.1 (4)	C53—C54—H54A	108.9
C20—C21—H21A	108.7	C55—C54—H54B	108.9
C22—C21—H21A	108.7	C53—C54—H54B	108.9
C20—C21—H21B	108.7	H54A—C54—H54B	107.7
C22—C21—H21B	108.7	C54—C55—C56	117.0 (3)
H21A—C21—H21B	107.6	C54—C55—H55A	108.0
C23—C22—C21	115.1 (4)	C56—C55—H55A	108.0
C23—C22—H22A	108.5	C54—C55—H55B	108.0
C21—C22—H22A	108.5	C56—C55—H55B	108.0
C23—C22—H22B	108.5	H55A—C55—H55B	107.3
C21—C22—H22B	108.5	C57—C56—C55	110.2 (3)
H22A—C22—H22B	107.5	C57—C56—H56A	109.6
C22—C23—C24	114.5 (4)	C55—C56—H56A	109.6
C22—C23—H23A	108.6	C57—C56—H56B	109.6
C24—C23—H23A	108.6	C55—C56—H56B	109.6
C22—C23—H23B	108.6	H56A—C56—H56B	108.1
C24—C23—H23B	108.6	C56—C57—N2	117.7 (3)
H23A—C23—H23B	107.6	C56—C57—H57A	107.9
C23—C24—C25	114.2 (3)	N2—C57—H57A	107.9
C23—C24—H24A	108.7	C56—C57—H57B	107.9
C25—C24—H24A	108.7	N2—C57—H57B	107.9
C23—C24—H24B	108.7	H57A—C57—H57B	107.2
C25—C24—H24B	108.7	N2—C58—H58A	109.5
H24A—C24—H24B	107.6	N2—C58—H58B	109.5
C24—C25—C26	114.3 (3)	H58A—C58—H58B	109.5
C24—C25—H25A	108.7	N2—C58—H58C	109.5
C26—C25—H25A	108.7	H58A—C58—H58C	109.5
C24—C25—H25B	108.7	H58B—C58—H58C	109.5
C26—C25—H25B	108.7	N2—C59—H59A	109.5
H25A—C25—H25B	107.6	N2—C59—H59B	109.5
C27—C26—C25	113.0 (3)	H59A—C59—H59B	109.5
C27—C26—H26A	109.0	N2—C59—H59C	109.5
C25—C26—H26A	109.0	H59A—C59—H59C	109.5
C27—C26—H26B	109.0	H59B—C59—H59C	109.5
C25—C26—H26B	109.0	C61—C60—N2	113.9 (3)
H26A—C26—H26B	107.8	C61—C60—H60A	108.8
C28—C27—C26	114.1 (3)	N2—C60—H60A	108.8
C28—C27—H27A	108.7	C61—C60—H60B	108.8
C26—C27—H27A	108.7	N2—C60—H60B	108.8
C28—C27—H27B	108.7	H60A—C60—H60B	107.7
C26—C27—H27B	108.7	C62—C61—C66	119.0 (4)
H27A—C27—H27B	107.6	C62—C61—C60	120.9 (4)
C27—C28—C29	113.6 (3)	C66—C61—C60	120.0 (4)
C27—C28—H28A	108.8	C61—C62—C63	120.3 (4)
C29—C28—H28A	108.8	C61—C62—H62	119.9
C27—C28—H28B	108.8	C63—C62—H62	119.9
C29—C28—H28B	108.8	C64—C63—C62	121.1 (5)
H28A—C28—H28B	107.7	C64—C63—H63	119.4
C30—C29—C28	110.3 (3)	C62—C63—H63	119.4
C30—C29—H29A	109.6	C63—C64—C65	119.4 (4)
C28—C29—H29A	109.6	C63—C64—H64	120.3
C30—C29—H29B	109.6	C65—C64—H64	120.3
C28—C29—H29B	109.6	C64—C65—C66	119.5 (4)
H29A—C29—H29B	108.1	C64—C65—H65	120.3
C29—C30—N1	117.5 (3)	C66—C65—H65	120.3
C29—C30—H30A	107.9	C61—C66—C65	120.7 (4)
N1—C30—H30A	107.9	C61—C66—H66	119.7
C29—C30—H30B	107.9	C65—C66—H66	119.7
N1—C30—H30B	107.9	C32—N1—C31	108.8 (3)
H30A—C30—H30B	107.2	C32—N1—C33	111.6 (3)
N1—C31—H31A	109.5	C31—N1—C33	109.7 (3)
N1—C31—H31B	109.5	C32—N1—C30	110.6 (3)
H31A—C31—H31B	109.5	C31—N1—C30	110.7 (3)
N1—C31—H31C	109.5	C33—N1—C30	105.4 (3)
H31A—C31—H31C	109.5	C58—N2—C59	108.4 (3)
H31B—C31—H31C	109.5	C58—N2—C60	111.4 (3)
N1—C32—H32A	109.5	C59—N2—C60	110.1 (3)
N1—C32—H32B	109.5	C58—N2—C57	110.6 (3)
H32A—C32—H32B	109.5	C59—N2—C57	106.4 (3)
N1—C32—H32C	109.5	C60—N2—C57	109.8 (3)
			
S2—Ni1—S1—C1	2.25 (13)	S16—C10—C11—S17	−1.5 (5)
S7—Ni1—S1—C1	179.83 (13)	S19—C10—C11—S17	178.6 (2)
S6—Ni1—S1—C1	18.6 (16)	S16—C10—C11—S18	176.4 (2)
S7—Ni1—S2—C2	−123.9 (9)	S19—C10—C11—S18	−3.5 (4)
S6—Ni1—S2—C2	178.06 (14)	Ni2—S17—C11—C10	1.0 (4)
S1—Ni1—S2—C2	−2.43 (14)	Ni2—S17—C11—S18	−176.8 (2)
S2—Ni1—S6—C4	175.20 (13)	C12—S18—C11—C10	1.1 (4)
S7—Ni1—S6—C4	−2.38 (13)	C12—S18—C11—S17	178.9 (3)
S1—Ni1—S6—C4	158.8 (15)	C10—S19—C12—S20	176.4 (3)
S2—Ni1—S7—C5	−56.2 (9)	C10—S19—C12—S18	−3.2 (3)
S6—Ni1—S7—C5	1.60 (13)	C11—S18—C12—S20	−177.8 (3)
S1—Ni1—S7—C5	−177.85 (13)	C11—S18—C12—S19	1.7 (3)
S12—Ni2—S11—C7	2.06 (14)	C13—C14—C15—C16	−180.0 (4)
S17—Ni2—S11—C7	−176.71 (14)	C14—C15—C16—C17	−179.4 (4)
S16—Ni2—S11—C7	97.1 (16)	C15—C16—C17—C18	−179.7 (4)
S11—Ni2—S12—C8	−1.66 (14)	C16—C17—C18—C19	179.1 (4)
S17—Ni2—S12—C8	72 (2)	C17—C18—C19—C20	−178.8 (4)
S16—Ni2—S12—C8	180.00 (13)	C18—C19—C20—C21	177.9 (4)
S11—Ni2—S16—C10	85.7 (16)	C19—C20—C21—C22	−178.2 (4)
S12—Ni2—S16—C10	−179.14 (14)	C20—C21—C22—C23	175.6 (4)
S17—Ni2—S16—C10	−0.37 (14)	C21—C22—C23—C24	179.6 (4)
S11—Ni2—S17—C11	−178.54 (14)	C22—C23—C24—C25	178.3 (4)
S12—Ni2—S17—C11	107 (2)	C23—C24—C25—C26	176.8 (4)
S16—Ni2—S17—C11	−0.21 (14)	C24—C25—C26—C27	−176.5 (4)
Ni1—S1—C1—C2	−1.6 (3)	C25—C26—C27—C28	176.4 (4)
Ni1—S1—C1—S4	178.90 (19)	C26—C27—C28—C29	−175.0 (4)
C3—S4—C1—C2	−0.1 (3)	C27—C28—C29—C30	−178.2 (3)
C3—S4—C1—S1	179.5 (2)	C28—C29—C30—N1	−176.8 (3)
S1—C1—C2—S2	−0.4 (5)	N1—C33—C34—C35	−89.3 (5)
S4—C1—C2—S2	179.1 (2)	N1—C33—C34—C39	95.5 (4)
S1—C1—C2—S3	−179.56 (19)	C39—C34—C35—C36	−0.5 (6)
S4—C1—C2—S3	0.0 (4)	C33—C34—C35—C36	−175.9 (4)
Ni1—S2—C2—C1	2.2 (4)	C34—C35—C36—C37	−0.6 (7)
Ni1—S2—C2—S3	−178.7 (2)	C35—C36—C37—C38	0.4 (7)
C3—S3—C2—C1	0.1 (3)	C36—C37—C38—C39	0.8 (7)
C3—S3—C2—S2	−179.0 (2)	C35—C34—C39—C38	1.7 (6)
C2—S3—C3—S5	179.0 (3)	C33—C34—C39—C38	177.0 (4)
C2—S3—C3—S4	−0.1 (2)	C37—C38—C39—C34	−1.9 (6)
C1—S4—C3—S5	−179.0 (3)	C40—C41—C42—C43	−178.7 (4)
C1—S4—C3—S3	0.1 (2)	C41—C42—C43—C44	179.5 (4)
Ni1—S6—C4—C5	3.0 (3)	C42—C43—C44—C45	−179.3 (4)
Ni1—S6—C4—S9	−177.27 (19)	C43—C44—C45—C46	−180.0 (4)
C6—S9—C4—C5	0.0 (3)	C44—C45—C46—C47	178.7 (4)
C6—S9—C4—S6	−179.7 (2)	C45—C46—C47—C48	179.1 (4)
S6—C4—C5—S7	−2.1 (5)	C46—C47—C48—C49	177.2 (4)
S9—C4—C5—S7	178.22 (19)	C47—C48—C49—C50	178.8 (4)
S6—C4—C5—S8	178.40 (19)	C48—C49—C50—C51	176.7 (4)
S9—C4—C5—S8	−1.3 (4)	C49—C50—C51—C52	−179.5 (4)
Ni1—S7—C5—C4	−0.1 (3)	C50—C51—C52—C53	179.5 (4)
Ni1—S7—C5—S8	179.34 (19)	C51—C52—C53—C54	174.1 (4)
C6—S8—C5—C4	1.9 (3)	C52—C53—C54—C55	179.9 (4)
C6—S8—C5—S7	−177.6 (2)	C53—C54—C55—C56	174.6 (4)
C5—S8—C6—S10	176.4 (3)	C54—C55—C56—C57	169.8 (4)
C5—S8—C6—S9	−1.9 (2)	C55—C56—C57—N2	151.0 (4)
C4—S9—C6—S10	−177.0 (3)	N2—C60—C61—C62	85.7 (4)
C4—S9—C6—S8	1.3 (2)	N2—C60—C61—C66	−93.6 (4)
Ni2—S11—C7—C8	−2.2 (4)	C66—C61—C62—C63	2.0 (6)
Ni2—S11—C7—S14	179.5 (2)	C60—C61—C62—C63	−177.3 (4)
C9—S14—C7—C8	−2.6 (4)	C61—C62—C63—C64	−1.0 (6)
C9—S14—C7—S11	175.8 (3)	C62—C63—C64—C65	−0.3 (7)
S11—C7—C8—S12	1.0 (5)	C63—C64—C65—C66	0.6 (6)
S14—C7—C8—S12	179.5 (2)	C62—C61—C66—C65	−1.8 (6)
S11—C7—C8—S13	−176.1 (2)	C60—C61—C66—C65	177.6 (3)
S14—C7—C8—S13	2.3 (4)	C64—C65—C66—C61	0.5 (6)
Ni2—S12—C8—C7	0.8 (4)	C34—C33—N1—C32	55.3 (4)
Ni2—S12—C8—S13	177.7 (2)	C34—C33—N1—C31	−65.4 (4)
C9—S13—C8—C7	−0.8 (4)	C34—C33—N1—C30	175.4 (3)
C9—S13—C8—S12	−177.9 (2)	C29—C30—N1—C32	−59.1 (4)
C7—S14—C9—S15	−176.7 (3)	C29—C30—N1—C31	61.6 (4)
C7—S14—C9—S13	2.0 (3)	C29—C30—N1—C33	−179.9 (3)
C8—S13—C9—S15	177.7 (3)	C61—C60—N2—C58	−63.5 (4)
C8—S13—C9—S14	−1.0 (3)	C61—C60—N2—C59	56.9 (4)
Ni2—S16—C10—C11	1.1 (4)	C61—C60—N2—C57	173.7 (3)
Ni2—S16—C10—S19	−179.0 (2)	C56—C57—N2—C58	−70.8 (5)
C12—S19—C10—C11	4.1 (3)	C56—C57—N2—C59	171.6 (4)
C12—S19—C10—S16	−175.8 (2)	C56—C57—N2—C60	52.5 (5)

## References

[bb1] Bruker (2000). *SMART*, *SAINT* and *SADABS* Bruker AXS Inc., Madison, Wisconsin, USA.

[bb2] Cassoux, P. (1999). *Coord. Chem. Rev.* **185–186**, 213–232.

[bb3] Cassoux, P., Valade, L., Kobayashi, H., Kobayashi, A., Clark, R. A. & Underhill, A. (1991). *Coord. Chem. Rev.* **110**, 115–160.

[bb4] Sheldrick, G. M. (2008). *Acta Cryst.* A**64**, 112–122. 10.1107/S0108767307043930 18156677

[bb5] Xue, G., Xu, W., Yu, W.-T. & Fang, Q. (2003). *Acta Cryst.* C**59**, m27–m29. 10.1107/s0108270102020115 12506220

